# Identification of two HLA-A*0201 immunogenic epitopes of lactate dehydrogenase C (LDHC): potential novel targets for cancer immunotherapy

**DOI:** 10.1007/s00262-020-02480-4

**Published:** 2020-01-13

**Authors:** Remy Thomas, Hibah Shaath, Adviti Naik, Salman M. Toor, Eyad Elkord, Julie Decock

**Affiliations:** Cancer Research Center, Qatar Biomedical Research Institute (QBRI), Hamad Bin Khalifa University (HBKU), Qatar Foundation (QF), Doha, Qatar

**Keywords:** LDHC, Lactate dehydrogenase, Cancer testis antigen, Epitopes, Adoptive T cell therapy, Cancer immunotherapy

## Abstract

Lactate dehydrogenase C (LDHC) is an archetypical cancer testis antigen with limited expression in adult tissues and re-expression in tumors. This restricted expression pattern together with the important role of LDHC in cancer metabolism renders LDHC a potential target for immunotherapy. This study is the first to investigate the immunogenicity of LDHC using T cells from healthy individuals. LDHC-specific T cell responses were induced by in vitro stimulation with synthetic peptides, or by priming with autologous peptide-pulsed dendritic cells. We evaluated T cell activation by IFN-γ ELISpot and determined cytolytic activity of HLA-A*0201-restricted T cells in breast cancer cell co-cultures. In vitro T cell stimulation induced IFN-γ secretion in response to numerous LDHC-derived peptides. Analysis of HLA-A*0201 responses revealed a significant T cell activation after stimulation with peptide pools 2 (PP2) and 8 (PP8). The PP2- and PP8-specific T cells displayed cytolytic activity against breast cancer cells with endogenous LDHC expression within a HLA-A*0201 context. We identified peptides LDHC^41−55^ and LDHC^288−303^ from PP2 and PP8 to elicit a functional cellular immune response. More specifically, we found an increase in IFN-γ secretion by CD8 + T cells and cancer-cell-killing of HLA-A*0201/LDHC positive breast cancer cells by LDHC^41−55^- and LDHC^288−303^-induced T cells, albeit with a possible antigen recognition threshold. The majority of induced T cells displayed an effector memory phenotype. To conclude, our findings support the rationale to assess LDHC as a targetable cancer testis antigen for immunotherapy, and in particular the HLA-A*0201 restricted LDHC^41–55^ and LDHC^288–303^ peptides within LDHC.

**Electronic supplementary material:**

The online version of this article (10.1007/s00262-020-02480-4) contains supplementary material, which is available to authorized users.

## Introduction

One of the major challenges in cancer immunotherapy remains the persistence of high affinity T cells specifically targeting tumor-associated antigens within an immunosuppressive tumor microenvironment. A good candidate target for immunotherapy should confer a high tumor selectivity with minimal adverse events. In addition, the target should preferably play a pivotal role in promoting tumor development and progression and/or impairing anti-tumor immunity, hence increasing the success rate of the therapeutic intervention to eradicate the tumor. Based on these criteria, the cancer testis antigen (CTA) lactate dehydrogenase C (LDHC) could be considered a novel promising immunotherapeutic target.

LDHC belongs to the lactate dehydrogenase family that catalyzes the interconversion of pyruvate and l-lactate and plays important roles in aerobic glycolysis [1]. Lactate dehydrogenase isozymes exist as homo- or hetero-tetramers composed of two major subunits, LDH-M and LDH-H that are encoded by *LDHA* and *LDHB,* respectively. Different combinations of these subunits assemble into 5 distinct isozymes with different tissue specificity; LDH1/LDHB (4H), LDH2 (3H1M), LDH3 (2H2M), LDH4 (1H3M) and LDH5/LDHA (4M). While LDHA is predominantly expressed in skeletal muscle and preferentially converts pyruvate to lactate, LDHB is mainly expressed in the heart and brain where it catalyzes the interconversion of lactate to pyruvate. LDHC, encoded by the *LDHC* gene, assembles into a homotetramer of LDHC subunits, also known as the LDHC or LDHX isoform [2]. Gene evolution models indicate that LDHC arose from gene duplication of the *LDHA* gene in mammals with 75% sequence homology with LDHA and 70% with LDHB [2]. LDHC expression is restricted to mature testis and spermatozoa, with low expression in oocytes and early embryos [3]. LDHC deficiency has been linked to male infertility, partly caused by diminished spermatozoa motility, whereas female *null* mice are fertile [4, 5]. Hence, the role of LDHC in spermatogenesis, oogenesis, fertility and early development remains unclear.

Although LDHC expression is tightly controlled and suppressed in normal somatic tissues, it is re-expressed in various malignant tissues, making its expression highly tumor specific [6]. Furthermore, increased LDHC expression has been associated with poor prognosis in renal cell carcinoma [7]. Very little data are available on the role of LDHC in cancer. Based on the observations of LDHA- and LDHB-mediated cancer progression, we can speculate that LDHC could be involved in metabolic reprograming of cancer cells. It is well established that growing tumors can bypass oxidative phosphorylation in favor of aerobic glycolysis to support their increasing metabolic need, which involves metabolic enzymes such as lactate dehydrogenases [8]. Indeed, dysregulation of LDHA and LDHB expression has been observed in tumors with increased glycolysis [9]. Hence, altered expression of LDHC could be involved in maintaining an alternative energy source by contributing to the metabolic switch in cancer cells. In addition, increased LDHA and decreased LDHB expressions facilitate tumor formation and progression through remodeling of the tumor microenvironment, increasing proliferation, and inducing epithelial-to-mesenchymal transition, cell migration and invasion, and angiogenesis [10–20]. In line with this, two studies to date demonstrate that enhanced expression of LDHC induces epithelial-to-mesenchymal transition, matrix metalloproteinase-9 (MMP9) expression and promotes cancer cell migration and invasion [7, 21].

Targeting LDHC could be a promising novel approach for cancer immunotherapy. First, given its restricted expression profile, it is likely that LDHC-specific immune-based interventions will result in the generation of LDHC-specific T cells with high affinity and low off-target effects. Moreover, targeting LDHC would not only inhibit LDHC-mediated cancer progression and specifically eradicate LDHC positive tumor cells, but could also induce reversal of the acidic tumor microenvironment, thereby releasing anti-tumor immunity. It is important to note that lactate and the concomitant tumor acidity negatively influence the anti-tumor immune response by skewing the immune cell compartment towards an immunosuppressive environment [22–24]. More specifically, LDHA has been found to promote upregulation of PD-L1 on tumor cells, impeding effector T cell activity [25]. Furthermore, elevated serum LDHA levels are associated with tumor burden as well as poor clinical outcome to PD-1 and CTLA-4 immune checkpoint blockade therapy [26]. Therefore, targeting key players of lactate metabolism including LDHC could aid to re-establish anti-tumor immunity and is a largely unexplored area of research.

In the present study, we generated several T cell responses against LDHC using either in vitro stimulation of T cells with synthetic peptides or priming of T cells with autologous peptide-pulsed dendritic cells. Using both approaches, we found that several peptide pools and individual peptides could elicit a cellular immune response, as determined by IFN-γ secretion. More in-depth analysis of the responses in HLA-A*0201 healthy donors enabled us to identify two HLA-A*0201-restricted immunogenic epitopes, LDHC^41−55^(LKDLADELALVDVAL) and LDHC^288−303^ (LSIPCVLGRNGVSDV), that could possibly be targeted by adoptive T cell therapy. Using different breast cancer cell lines, we demonstrated that LDHC^41−55^and LDHC^288−303^ specific T cells were capable of recognizing and eradicating HLA-A*0201 positive/LDHC positive tumor cells, while no specific cytolytic activity was detected against HLA-A*0201 negative/LDHC positive tumor cells. Interestingly, we found that reduction of LDHC expression in the HLA-A*0201 positive cancer cells attenuated the T cell responses against LDHC, suggesting a plausible threshold of LDHC expression to elicit immune reactivity. To conclude, we demonstrate for the first time that LDHC exhibits immunogenicity and our findings warrant further study into the potential of LDHC as a novel therapeutic target for cancer immunotherapy.

## Materials and methods

### Cell culture

MDA-MB-453, MDA-MB-468, and MDA-MB-231 breast cancer cells were maintained in DMEM (Gibco-BRL, Waltham, MA, USA) supplemented with 10% (v/v) FBS (Hyclone US origin, GE Healthcare Lifesciences, Pittsburg, PA, USA), 50 U/ml penicillin and 50 μg/ml streptomycin (Gibco-BRL, Waltham, MA, USA). MDA-MB-436 breast cancer cells were maintained in DMEM (Gibco-BRL, Waltham, MA, USA) supplemented with 10% (v/v) FBS (Hyclone US origin, GE Healthcare Lifesciences, Pittsburg, PA, USA), 50 U/ml penicillin and 50 μg/ml streptomycin (Gibco-BRL, Waltham, MA, USA), 10 mg/ml insulin and 16 mg/ml glutathione (Sigma-Aldrich, St. Louis, MO, USA). BT549 breast cancer cells were maintained in American Tissue Culture Collection (ATCC)-formulated RPMI-1640 medium (Gibco-BRL, Waltham, MA, USA) supplemented with 10% (v/v) FBS (Hyclone US origin, GE Healthcare Lifesciences, Pittsburg, PA, USA), 50 U/ml penicillin and 50 μg/ml streptomycin (Gibco-BRL, Waltham, MA, USA), and 0.023 IU/ml insulin (Sigma-Aldrich, St. Louis, MO, USA). HCC1500 breast cancer cells and T2 cells were maintained in ATCC-formulated RPMI-1640 medium (Gibco-BRL, Waltham, MA, USA) supplemented with 10% (v/v) FBS (Hyclone US origin, GE Healthcare Lifesciences, Pittsburg, PA, USA), 50 U/ml penicillin and 50 μg/ml streptomycin (Gibco-BRL, Waltham, MA, USA). All cell lines were maintained at 37 °C, 5% CO_2_ and regular mycoplasma testing was performed using a PCR-based assay of culture supernatants (Forward primer 5′-gggagcaaacaggattagataccct-3′ and reverse primer 5′-tgcaccatctgtcactctgttaacctc-3′).

### LDHC breast cancer cell lines

Adherent HCC1500 and BT549 cells were transduced at 80% confluency with purified GFP-positive shLDHC lentiviral particles (SMARTvector Lentiviral Human LDHC hCMV-TurboGFP shRNA, #V3SH11240-229943916, Dharmacon, Lafayette, CO, USA) or purified GFP-positive negative control shCTR lentiviral particles (SMARTvector non-targeting hCMV-TurboGFP control particles, #S-005000-01, Dharmacon, Lafayette, CO, USA). After 6 days, transduced HCC1500 and BT549 cells were maintained under 0.5 ug/ml puromycin (Sigma-Aldrich, St. Louis, MO, USA) selection. In addition, the MDA-MB-468 cell line was transduced with the GFP positive shCTR lentiviral particles.

### LDHC peptides

A synthetic peptide library consisting of 81 individual 15-mer peptides with a 11-residue overlap was purchased as a custom-made service (JPT Peptide Technologies, Berlin, Germany). Individual peptides were reconstituted at 2 ug/ul in DMSO. Peptide pools of 10 (peptide pools 1–7) or 11 peptides (peptide pool 8) were generated with a final concentration of 200 ug/ml of each individual peptide or 2 mg/ml total peptide content (Table 1). Finally, peptides were used at 2 ug/ml to stimulate dendritic cells or T cells.

**Table 1:** LDHC synthetic peptide library

	ID	Sequence		ID	Sequence
PP1	P1	H-MSTVKEQLIEKLIED-OH	PP5	P41	H-SGCNLDSARFRYLIG-OH
	P2	H-KEQLIEKLIEDDENS-OH		P42	H-LDSARFRYLIGEKLG-OH
	P3	H-IEKLIEDDENSQCKI-OH		P43	H-RFRYLIGEKLGVHPT-OH
	P4	H-IEDDENSQCKITIVG-OH		P44	H-LIGEKLGVHPTSCHG-OH
	P5	H-ENSQCKITIVGTGAV-OH		P45	H-KLGVHPTSCHWIIG-OH
	P6	H-CKITIVGTGAVGMAC-OH		P46	H-HPTSCHGWIIGEHGD-OH
	P7	H-IVGTGAVGMACAISI-OH		P47	H-CHGWIIGEHGDSSVP-OH
	P8	H-GAVGMACAISILLKD-OH		P48	H-IIGEHGDSSVPLWSG-OH
	P9	H-MACAISILLKDLADE-OH		P49	H-HGDSSVPLWSGVNVA-OH
	P10	H-ISILLKDLADELALV-OH		P50	H-SVPLWSGVNVAGVAL-OH
PP2	**P11**	**H-LKDLADELALVDVAL-OH**	PP6	P51	H-WSGVNVAGVALKTLD-OH
	P12	H-ADELALVDVALDKLK-OH		P52	H-NVAGVALKTLDPKLG-OH
	P13	H-ALVDVALDKLKGEMM-OH		P53	H-VALKTLDPKLGTDSD-OH
	P14	H-VALDKLKGEMMDLQH-OH		P54	H-TLDPKLGTDSDKEHW-OH
	P15	H-KLKGEMMDLQHGSLF-OH		P55	H-KLGTDSDKEHWKNIH-OH
	P16	H-EMMDLQHGSLFFSTS-OH		P56	H-DSDKEHWKNIHKQVI-OH
	P17	H-LQHGSLFFSTSKITS-OH		P57	H-EHWKNIHKQVIQSAY-OH
	P18	H-SLFFSTSKITSGKDY-OH		P58	H-NIHKQVIQSAYEIIK-OH
	P19	H-STSKITSGKDYSVSA-OH		P59	H-QVIQSAYEIIKLKGY-OH
	P20	H-ITSGKDYSVSANSRI-OH		P60	H-SAYEIIKLKGYTSWA-OH
PP3	P21	H-KDYSVSANSRIVIVT-OH	PP7	P61	H-IIKLKGYTSWAIGLS-OH
	P22	H-VSANSRIVIVTAGAR-OH		P62	H-KGYTSWAIGLSVMDL-OH
	P23	H-SRIVIVTAGARQQEG-OH		P63	H-SWAIGLSVMDLVGSI-OH
	P24	H-IVTAGARQQEGETRL-OH		P64	H-GLSVMDLVGSILKNL-OH
	P25	H-GARQQEGETRLALVQ-OH		P65	H-MDLVGSILKNLRRVH-OH
	P26	H-QEGETRLALVQRNVA-OH		P66	H-GSILKNLRRVHPVST-OH
	P27	H-TRLALVQRNVAIMKS-OH		P67	H-KNLRRVHPVSTMVKG-OH
	P28	H-LVQRNVAIMKSIIPA-OH		P68	H-RVHPVSTMVKGLYGI-OH
	P29	H-NVAIMKSIIPAIVHY-OH		P69	H-VSTMVKGLYGIKEEL-OH
	P30	H-MKSIIPAIVHYSPDC-OH		P70	H-VKGLYGIKEELFLSI-OH
PP4	P31	H-IPAIVHYSPDCKILV-OH	PP8	P71	H-YGIKEELFLSIPCVL-OH
	P32	H-VHYSPDCKILVVSNP-OH		P72	H-EELFLSIPCVLGRNG-OH
	P33	H-PDCKILVVSNPVDIL-OH		**P73**	**H-LSIPCVLGRNGVSDV-OH**
	P34	H-ILVVSNPVDILTYIV-OH		P74	H-CVLGRNGVSDVVKIN-OH
	P35	H-SNPVDILTYIVWKIS-OH		P75	H-RNGVSDVVKINLNSE-OH
	P36	H-DILTYIVWLISGLPV-OH		P76	H-SDVVKINLNSEEEAL-OH
	P37	H-YIVWKISGLPVTRVI-OH		P77	H-KINLNSEEEALFKKS-OH
	P38	H-KISGLPVTRVIGSGC-OH		P78	H-NSEEEALFKKSAETL-OH
	P39	H-LPTVRVIGSGCNLDS-OH		P79	H-EALFKKSAETLWNIQ-OH
	P40	H-RVIGSGCNLDSARFR-OH		P80	H-KKSAETLWNIQKDLI-OH
				P81	H-KSAETLWNIQKDLIF-OH

A custom-made 15-mer LDHC peptide library was established, containing 81 peptides with an 11-residue overlap

P11 and P73, highlighted in bold, were identified as immunogenic HLA-A*0201-restricted epitopes

### Blood samples and peripheral blood mononuclear cell isolation

Peripheral blood mononuclear cells (PBMCs) were isolated from buffy coat samples from 14 healthy individuals visiting the blood donation unit at Hamad Medical Corporation. Buffy coat samples were diluted five times with Dulbecco’s Phosphate-Buffered Saline (Gibco-BRL, Waltham, MA, USA), after which 10 ml was layered on top of 10 ml Lymphoprep™ (Stem Cell Technologies, Vancouver, Canada) followed by separation into layers by density gradient centrifugation. The interphase containing the PBMCs was carefully collected and transferred to new tubes and washed twice with serum free RPMI-1640 media (Gibco-BRL, Waltham, MA, USA). The cells were counted and 10 × 10^6^ PBMCs were frozen per vial. On average, 500 × 10^6^ PBMCs were isolated with > 90% cell viability after cryopreservation. HLA typing of PBMCs from all 14 healthy individuals was performed at the Department of Laboratory Medicine & Pathology, Hamad Medical Corporation. HLA typing was obtained for nine different HLA loci (A, B, C, DRB1, DRB3/4/5, DQA1, DQB1, DPA1, and DPB1) and the information on class I alleles is summarized in Table 2.

**Table 2:** Demographics of study cohort

Donor	Sex	Ethnicity	HLA class I	P11 predicted binding (9-mer/12-mer)		P73 predicted binding (9-mer/12-mer)	
				A allele	B allele	A allele	B allele
D02	M	Arab	A*33, B*14, C*08	NA	21/18	NA	11/16
D03	F	Arab	A*01, B*35, C*04	16/14	14/13	10/8	11/10
D04	F	Arab	A*03, B*41, C*03	18/22	24/15	22/23	4/2
D05	F	Filipina	A*11, B*07, C*01	11/15	15/14	25/16	12/18
D06	F	Asian	A*24, B*18, C*07	13/12	18/16	04/03	2/5
D07	M	Asian	A*01, B*35, C*04	16/14	14/13	10/8	11/10
D08	F	Asian	A*24, B*15, C*03	13/12	17/13	04/03	18/12
D09	M	Arab	A*02, B*18, C*07	28/24	18/16	18/25	2/5
D10	M	Arab	A*23, B*27, C*02	NA	16/12	NA	15/6
D11	M	Asian	A*02, B*35, C*03	28/24	14/13	18/25	11/10
D12	F	Arab	A*03, B*39, C*12	18/22	15/21	22/23	8/7
D13	M	Arab	A*30, B*15, C*17	NA	17/13	NA	18/12
D14	F	Asian	A*02, B*15, C*04	28/24	17/13	18/25	18/12
D15	F	Arab	A*02, B*50, C*06	28/24	20/10	18/25	1/1

For each allele, the highest prediction score is depicted

*M* male, *F* Female

### In vitro stimulation (IVS) of T lymphocytes

PBMCs were seeded at 2 × 10^5^ cells/well in 96-well U bottom plates in complete RPMI-1640 medium (Gibco-BRL, Waltham, MA, USA), containing either 20 ug/ml individual peptide, 20ug/ml peptide pool or no peptides (control). Every 2 days, half of the medium was replenished with complete RPMI-1640 medium (Gibco-BRL, Waltham, MA, USA), supplemented with 250 IU/ml of IL-2 (rhIL-2, #202-IL-050/CF, RnD systems, Minneapolis, MN, USA), and 50 ng/ml of IL-15 (rhIL15, #247-ILB-025/CF, RnD systems, Minneapolis, MN, USA). After 18 days, LDHC-specific T cell responses were determined by IFN-γ ELISpot assay.

### Differentiation and maturation of autologous dendritic cells (DCs)

PBMCs from HLA-A*0201 positive healthy individuals were used for the differentiation and maturation of dendritic cells. PBMCs were seeded at a density of 5 × 10^6^/well in a 24-well plate and after 2 h the non-adherent fraction (peripheral blood lymphocytes) was removed and cryopreserved for future incubation with mature autologous DCs. Differentiation of the adherent cells into dendritic cells was induced by GM-CSF (1000U/ml, #300-03, PeproTech, Rocky Hill, NJ, USA) and IL-4 (1000U/ ml; #200-04, PeproTech, Rocky Hill, NJ, USA) with replenishment every 2 days. On day 5, maturation of 0.5 × 10^6^ DCs was induced using 100 ng/ml LPS (Sigma-Aldrich, St. Louis, MO, USA) and maturation was checked on day 8 by flow cytometry (BD LSRFortessa X-20; Software Diva) using markers for CD83 (Anti-Human CD83 APC; #551073, BD Biosciences, Franklin Lakes, NJ, USA) & CD86 (Anti-Human CD86 FITC; #555,657, BD Biosciences, Franklin Lakes, NJ, USA).

### Generation of LDHC-specific T cells by dendritic cell stimulation

Mature dendritic cells (1 × 10^5^) were pulsed with either 20 ug/ml individual peptide, 20 ug/ml peptide pool or no peptides (control) for 2 h at 37 °C. Next, peptide-pulsed DCs were used to prime the previously cryopreserved autologous peripheral blood lymphocytes (PBL) in a 96-well U bottom plate at a DC:PBL ratio of 1:20 (25,000 DCs:500,000 PBLs) using complete RPMI-1640 media (Gibco-BRL, Waltham, MA, USA), supplemented with 250 IU/ml rhIL-2 (rhIL-2, #202-IL-050/CF, RnD systems, Minneapolis, MN, USA) and 50 ng/ml rhIL-15 (rhIL15, #247-ILB-025/CF, RnD systems, Minneapolis, MN, USA). Half of the feeding medium was replenished every 2 days. After 7 days, PBLs were re-stimulated with freshly pulsed autologous DCs for another 7 days. After the second cycle of priming, T cells were collected for functional analyses (IFN-γ ELISpot or co-culture with breast cancer cells).

### T2 cell loading assay

HLA-A*02 specificity of peptides P11 and P73 was determined using loaded T2 cells as antigen presenting cells in co-culture with primed T cells. T2 cells were loaded with 20 ug/ml of P11, P73, the non-reactive peptide P78 peptide or no peptide (control) for 2 h. Peptide loaded-T2 cells were incubated overnight with their respective DC pulsed-T cells at an E:T ratio of 50:1, followed by measurement of the number of IFN-γ spot forming units (SFU) by ELISpot.

### Co-culture of expanded T cells with breast cancer cells

The cytotoxic ability of LDHC-specific T cells (HLA-A*0201 positive) was determined by co-culture with several breast cancer cell lines in comparison to control-T cells (no peptides). For this purpose, we used several breast cancer cell lines:MDA-MB-468 (HLA-A*0201 negative) cells with endogenous LDHC expression (A2−/high) and two LDHC loss-of-function HLA-A*0201 cell line models (HCC1500 and BT549) with endogenous LDHC expression and transduced with shCTR (A2 + /high) or transduced with shLDHC (A2 + /low). Co-cultures were maintained for 4 h at 37 °C in 96 well U-bottom plates at an E:T ratio of 50:1. Production of IFN-γ was determined by ELISpot and cytolytic activity was assessed by viability assay as described below.

### CD4 + T cell depletion

After co-culture of control- (no peptides), P11- and P73-specific T cells with A2 + /high HCC1500 cells, cells were subjected to CD4 + T cell depletion using human CD4 microbeads (#130–045-101, Miltenyi Biotec, Bergisch Gladbach, Germany) and the autoMACS Pro Separator (Miltenyi Biotec, Bergisch Gladbach, Germany), as per the manufacturer’s instructions. T cell reactivity of the CD4 + depleted and CD4 + fractions were determined by IFN-γ ELISpot.

### IFN-γ ELISpot

IFN-γ release was determined using the Human IFN-γ ELISpotPLUS HRP assay (#3420-4HST-10, Mabtech, Nacka Strand, Sweden) following the manufacturer’s guidelines. Wells were washed four times with PBS (Gibco-BRL, Waltham, MA, USA) and pre-conditioned with complete RPMI-1640 media (Gibco-BRL, Waltham, MA, USA) for 30 min. Expanded T cells were seeded in the wells at 5 × 10^4^ with either 20 ug/ml individual peptide, 20 ug/ml peptide pool, no peptides (control) or anti-human anti-CD3 antibody as a positive control (mAb CD3-2, #3420-4HST-10, Mabtech, Nacka Strand, Sweden) and left to incubate overnight at 37 °C. The number of IFN-γ SFUs was determined using the AID iSpot ELISpot reader (Autoimmun Diagnostika GmbH, Strasburg, Germany).

### Viability assay

Following 4 h of co-culture, all cells were collected, washed with PBS, and stained with 7-Aminoactinomycin D (7-AAD) (#00–6993-50, eBiocience, San Diego, CA, USA) at 4 °C for 20 min in the dark. Next, cells were washed with PBS and resuspended in staining buffer (#554656, BD Biosciences, Franklin Lakes, NJ, USA). Within the GFP positive cancer cells, the percentage of 7-AAD positive cells was determined using the BD LSRFortessa X-20 instrument and FlowJo (BD Biosciences, Franklin Lakes, NJ, USA).

### RNA isolation and cDNA synthesis

Total RNA was isolated from the breast cancer cell lines using the PureLink RNA Mini kit (#12183018A, Ambion, Thermo Fisher Scientific, Waltham, MA, USA) following the manufacturer’s protocol. The RNA quantity and purity was assessed by A260/A230 and A260/A280 absorbance measurement (Nanodrop Technologies, Wilmington, DE, USA). Reverse transcription of 1 µg RNA was performed using Moloney Murine Leukemia Virus (MMLV)-Superscript (#28025013, Thermo Fisher Scientific, Waltham, MA, USA) and random hexamers (#SO142, Thermo Fisher Scientific, Waltham, MA, USA) resulting in a final concentration of 50 ng/µl cDNA.

### Quantitative real-time RT-PCR

Real time qRT-PCR was conducted using 50 ng cDNA and specific 5′FAM-3′MGB Taqman gene expression primer/probe sets to determine the mRNA expression of LDHC (Hs01022301_m1, Applied Biosystems, Foster City, CA, USA) and the housekeeping gene 60S acidic ribosomal protein P0 (RPLPO, #4333761F, Applied Biosystems, Foster City, CA, USA).

### LDHC western blot

Breast cancer cells were harvested at 80% confluency and lysed on ice using Radioimmunoprecipitation assay (RIPA) lysis buffer (#89900, Thermo Fisher Scientific, Waltham, MA, USA) containing Halt™ EDTA-free protease inhibitor cocktail mix (#78425, Thermo Fisher Scientific, Waltham, MA, USA). Cell lysates were centrifuged at 12,000 rpm for 25 min, supernatants were collected and total protein content was determined using the Bicinchoninic acid assay (BCA) protein assay (#23225, Thermo Fisher Scientific, Waltham, MA, USA). Protein samples were denatured in 4 × Laemmli sample buffer (#1610747, Bio-Rad Laboratories, Hercules, CA, USA) at 60 °C for 10 min and equal amounts of protein (80ug) were loaded onto a 4–15% Tris-Glycine eXtended (TGX) protein gel (#4561084, Bio-Rad Laboratories, Hercules, CA, USA). Proteins were transferred onto 0.2 µm polyvinylidinedifluoride membranes (#1704156, Bio-Rad Laboratories, Hercules, CA, USA) followed by blocking in 5% non-fat dried milk/Tris-buffered saline and 0.1% Tween-20 (TBST) for 1 h at room temperature. The membranes were incubated overnight at 4 °C with the following primary antibodies diluted in 5% non-fat dried milk/TBST: rabbit anti-LDHC (1:500, #ab52747, Abcam, Cambridge, UK) and rabbit anti-β-actin (clone 13E5, #4970, 1:1000, Cell Signaling technologies, Danvers, MA, USA). Membranes were washed three times each with TBST and Tris-buffered saline (TBS) for 5 min each and probed with horseradish peroxidase-conjugated goat anti-rabbit secondary antibodies (1:5000, # 111-035-003, Jackson ImmunoResearch, West Grove, PA, USA) for 1 h at room temperature followed by washes as before. Bound antibodies were detected using Enhanced ChemiLuminescence (ECL) Plus (#32132, Thermo Fisher Scientific, Waltham, MA, USA) or ECL Supersignal-West Femto (#34095, Thermo Fisher Scientific, Waltham, MA, USA) on the Chemidoc XRS + Imaging system (Bio-Rad Laboratories, Hercules, CA, USA). Images were acquired and processed with the Image Lab software (Bio-Rad Laboratories, Hercules, CA, USA).

### LDHC expression by flow cytometry

Flow cytometry was used to determine the protein expression of LDHC across breast cancer cell lines. Approximately 1 × 10^6^ cells were fixed and permeabilised (#554714, BD Biosciences, Franklin Lakes, NJ, USA), and resuspended in 100 µL of staining buffer containing 2.5 µg of Human BD Fc Block™ (#130-059-901, Miltenyi Biotec, Bergisch Gladbach, Germany). After 10 min at room temperature, the cells were incubated with anti-human LDHC antibody (#ab52747, Abcam, Cambridge, UK) at a concentration of 1:50 for one hour followed by two washes with PBS. Next, anti-rabbit secondary antibody (Alexa-Flour 647, #A31573, Thermo Fisher Scientific, Waltham, MA, USA) was added at a concentration of 1:1000 for 1 h followed by two washes with PBS. LDHC expression was analyzed using the BD LSRFortessa X-20 instrument and FlowJo (BD Biosciences, Franklin Lakes, NJ, USA).

### Immunophenotyping of generated T cells

Multiparametric flow cytometry was performed to characterize the generated T cell responses after peptide- or control (no peptides)-stimulation or priming with peptide- or control (no peptide)-pulsed DCs. T cells were washed and resuspended in 100 µL of staining buffer containing 2.5 µg of Human BD Fc Block™ (#130-059-901, Miltenyi Biotec, Bergisch Gladbach, Germany). Cell surface staining of various markers was obtained using the following antibodies: CD3-APC-Cy7 (#560176; clone SK7; BD Biosciences, Franklin Lakes, NJ, USA), CD4-PE-efluor 610 (#61-0049-42; clone RPA-T4; eBioscience, San Diego, CA, USA), CD45RA-APC (#304112; clone HI100; BioLegend, San Diego, CA, USA), CD45RO-BUV395 (#564291, clone UCHL1, BD Biosciences, Franklin Lakes, NJ, USA), CD62L-BV786 (#565312, clone SK11, BD Biosciences, Franklin Lakes, NJ, USA) and CCR7-BV711 (#563712, clone 3D12, BD Biosciences, Franklin Lakes, NJ, USA). Dead cells were gated out using the 7-AAD viability dye (#00-6993-50, eBiocience, San Diego, CA, USA). Data analysis was performed using the BD LSRFortessa X-20 instrument and FlowJo software (BD Biosciences, Franklin Lakes, NJ, USA).

### Statistics

Gaussian distribution of data was assessed using the Shapiro–Wilk test. Non-parametric analyses were conducted using Kruskal–Wallis test, while parametric analyses were performed using unpaired, 2-tailed *t* test or 1-way ANOVA. Data are represented as mean ± SEM unless stated otherwise.

## Results

### LDHC-specific T cell responses

**Fig. 1:**
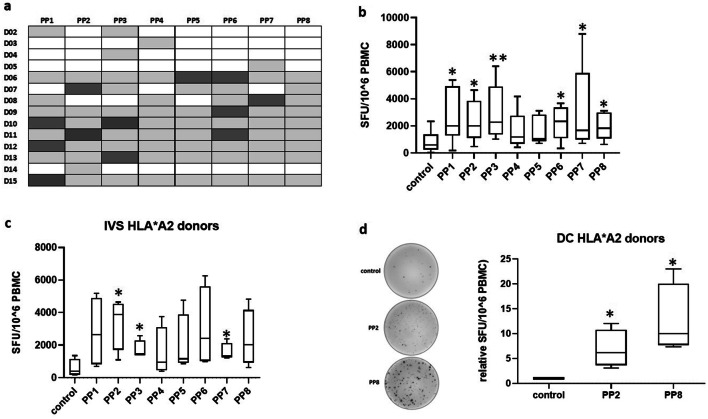
Detection of LDHC-specific T cell responses in vitro. PBMCs were isolated from healthy individuals and stimulated in vitro with peptide pools or without peptides (control), or were primed by autologous peptide- or control (no peptides)-pulsed dendritic cells. **a** T cell responses against LDHC peptide pools or control (no peptides) after in vitro stimulation, as determined by IFN-γ ELISpot. Positive T cell responses are highlighted in grey and were defined as SFU/10^6^ cells ≥ 100 with PP/control ratio ≥ 3. The strongest response for each donor is highlighted in dark grey. **b** PBMCs of 14 donors were stimulated with peptide poolsor control condition (no peptides) and supplemented with IL-2 and IL-15 for 18 days, followed by ELISpot to determine the number of IFN-γ spot forming units per 10^6^ PBMCs. Tukey box plot of positive T cell responses across all donors. **c** IFN-γ ELISpot after in vitro peptide-or control (no peptides)-stimulation of PBMCs of four HLA-A*0201 healthy individuals. **d** IFN-γ release of HLA-A*0201 T cells after 2 cycles of priming with autologous peptide- or control (no peptides)-pulsed DCs. Representative IFN-γ ELISpot images are given for one donor, while Tukey box plots represent data of all four donors. The control conditions are T cells stimulated with solvent only (no peptides). *p < 0.05, **p < 0.01

We investigated the immunogenicity of LDHC using a 15-mer synthetic peptide library of 8 peptide pools (PP1–PP8), each containing 10–11 individual peptides. LDHC-specific T cell responses were generated using either in vitro stimulation of peripheral blood mononuclear cells with overlapping peptide pools, or through two rounds of T cell priming with peptide pool-pulsed autologous dendritic cells. We observed a wide range of generated T cell responses, measured by IFN-γ release, upon in vitro stimulation of PBMCs from 14 healthy donors (D02 to D15). As summarized in Fig. 1a, each donor displayed positive responses (highlighted in grey) against several peptide pools with a positive T cell response defined as SFU/10^6^ cells ≥ 100 with PP/control ratio ≥ 3. Some of the peptide pools induced stronger responses across multiple donors compared to the other pools. For instance, PP1 and PP6 induced the strongest response across three donors (PP1: D10, D12, and D15; PP6: D06, D09, and D11), while PP2 and PP3 induced the greatest reactivity in two donors (PP2: D07 and D11; PP3: D10 and D13) **(**Fig. 1a). This suggests that some of the peptide pools may be more immunogenic and that HLA-restriction is shared across donors. Overall, we found a significant increase in IFN-γ secretion after stimulation with all peptide pools except for PP4 and PP5 (Fig. 1b). Next, we focused our analysis on healthy donors with the HLA-A*02 type as HLA-A2 is the most abundant HLA molecule in the European/North American Caucasian population (27%) as well as in the Arab population (25–30%) [27, 28]. More specifically, HLA-A*0201 allele frequency reached 28% in our study (4/14). Using in vitro stimulation of HLA-A*02 restricted T cells from four donors, we obtained positive T cell responses against all peptide pools with the strongest significant response generated against PP2 (5.9-fold, *p* = 0.016) (Fig. 1c). After priming the T cells with peptide-pulsed DCs, we obtained stronger responses with a significant induction of IFN-γ secretion against PP2 (6.9 fold, *p* = 0.021) and against PP8 (12.6-fold, *p* = 0.017) (Fig. 1d). As can be seen in the representative IFN-γ ELISpot images, PP8 induced a much stronger T cell response than PP2. Further experiments in this study were carried out using T cells from all four HLA-A*02 donors.

### Cytolytic activity of LDHC-specific HLA-A*0201 restricted T cells

**Fig. 2:**
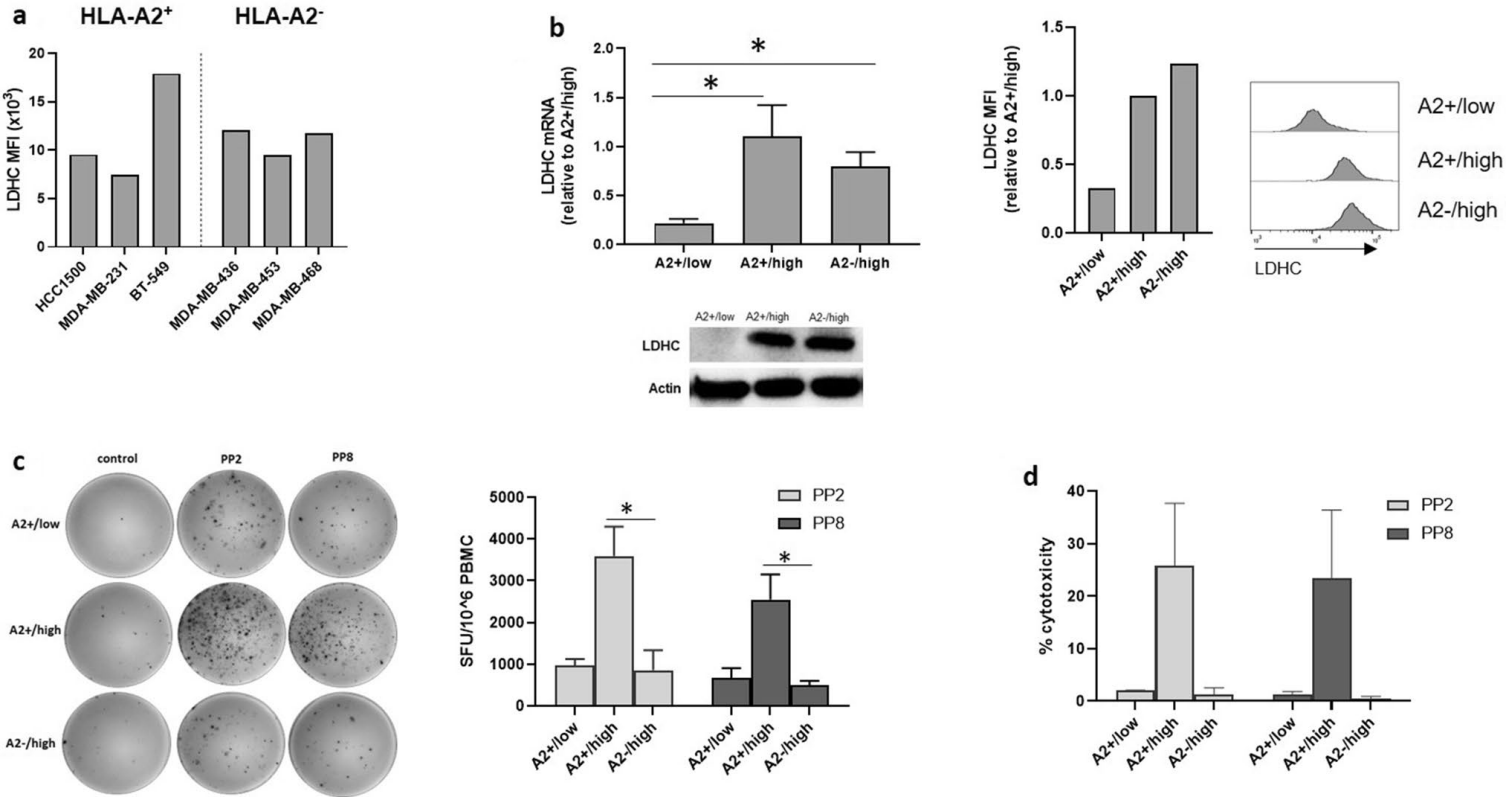
Cancer cell/immune cell co-culture experiments with HLA-A*0201- restricted T cells. LDHC-specific T cells, generated by priming of HLA-A*0201 T cells with peptide pool- or control (no peptides)-pulsed DCs, were co-cultured with various breast cancer cell lines, followed by IFN-γ ELISpot and cytotoxicity assays. **a** LDHC expression across a panel of HLA-A*02 positive and negative breast cancer cell lines, as determined by flow cytometry. **b** Endogenous LDHC expression level of one HLA-A*0201 negative (MDA-MB-468, defined as A2−/high) and one HLA-A*0201 positive (HCC1500, defined as A2 + /high) breast cancer cell line was assessed by qRT-PCR, western blotting and flow cytometry. In addition, LDHC expression was reduced by shRNA in the HLA-A*0201 cell line to obtain the A2 + /low cell line. **c** Number of IFN-γ spot forming units/10^6^ PBMCs and **d** cytolytic activity of PP2- and PP8-primed HLA-A*0201 T cells in co-culture with different breast cancer cell lines (A2 + /low, A2 + /high and A2−/high). Representative IFN-γ ELISpot images are given for one donor, while Tukey box plots represent data of all four donors. MFI, mean fluorescence intensity.*p < 0.05

Successful eradication of tumor cells by the immune system encompasses multiple steps including the ability of T cells to specifically recognize tumor cells due to dysregulated expression of tumor-associated antigens. Thus, we used flow cytometry to determine the expression of LDHC across a panel of breast cancer cell lines, including HLA-A2^−^ and HLA-A2^+^ cell lines (Fig. 2a). Based on this analysis, we selected HCC1500 as HLA-A2^+^ and MDA-MB-468 as HLA-A2^−^ cell line model with good expression of LDHC. In addition, we have used a second HLA-A2^+^ cell line, BT-549, to confirm some of our findings. To evaluate the cytolytic activity of LDHC-specific HLA-A*02-restricted T cells, we co-cultured T cells primed with peptide-pulsed DCs together with different breast cancer cell lines. We used shRNA to reduce the expression of LDHC in the HLA-A*0201 positive HCC1500 breast cancer cell line, resulting in A2 + /LDHC low cells with silenced LDHC expression (A2 + /low), in addition to the parental A2 + /LDHC high cells with endogenous high expression of LDHC (A2 + /high). We also included the MDA-MB-468 cell line with endogenous LDHC expression but lacking HLA-A*0201 as a negative control cell line (A2−/high).Using qRT-PCR, western blot and flow cytometry, we demonstrated the higher LDHC RNA and protein expression (mean fluorescence intensity of LDHC staining) in the A2 + /high and A2−/high cells as compared to the A2 + /low cells (Fig. 2b). Next, we primed T cells with autologous peptide-pulsed dendritic cells for 2 weeks after which the LDHC-specific T cells were incubated with the various breast cancer cell lines for 4 h. For this analysis, we focused on peptide pools PP2 and PP8 given their ability to induce HLA-A*02 restricted T cell activity (Fig. 1d). As shown in Fig. 2c, co-culture of the peptide-specific T cells with the A2 + /high cell line greatly induced IFN-γ secretion in comparison to the IFN-γ levels after incubation with the A2−/high cell line (PP2: 4.2-fold, *p* = 0.018; PP8: 5.2-fold, *p* = 0.015). In line with this finding, we found a strong increase in tumor cell killing of the A2 + /high cell line by PP2- (26% versus 1.4% in A2−/high) and PP8 specific T cells (24% versus 0.5% in A2−/high) (Fig. 2d). Moreover, the increased IFN-γ production and cytolytic activity could be reduced to similar levels observed in A2−/high co-cultures by reducing the expression of LDHC in the parental A2 + /high cell line thereby generating the HCC1500-derived cell line with A2 + /low phenotype (Fig. 2c, d).

### Identification of HLA-A*0201-restricted LDHC-derived immunogenic peptides

Next, we investigated which individual peptides within PP2 and PP8 could induce LDHC-specific T cell responses in HLA-A*0201 donors. We pulsed mature DCs within individual peptides from PP2and PP8 and used these to prime autologous T cells followed by co-culture with the aforementioned breast cancer cell lines.

**Fig. 3:**
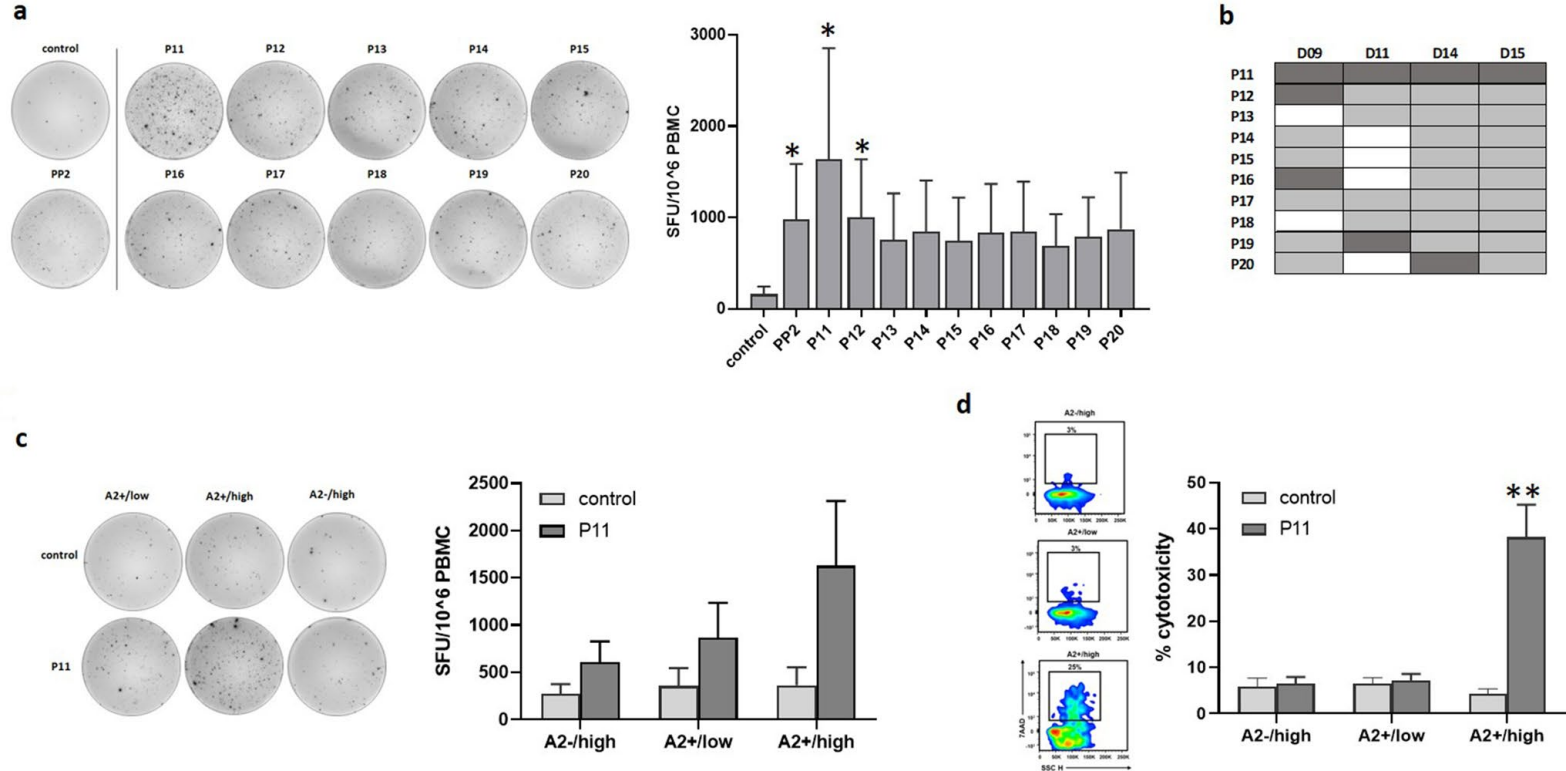
Identification of HLA-A*0201 T cell responses against individual peptides within PP2. Peptide-specific T cells were generated by priming of HLA-A*0201 T cells with DCs loaded with individual peptides P11–P20, or without peptides (control). **a** T cell responses generated against individual peptides of PP2 or control (no peptides). Representative IFN-γ ELISpot images are given for one donor, while Tukey box plots represent data of all four donors. **b** Overview of T cell responses against individual PP2-peptides or control (no peptides). Positive T cell responses are depicted in grey with the strongest response for each donor highlighted in dark grey. **c** IFN-γ secretion and **d** cytolytic activity of P11- or control (no peptides)-primed HLA-A*0201 T cells in co-culture with different breast cancer cell lines (A2 + /low, A2 + /high and A2−/high). Representative IFN-γ ELISpot images and density plots are given for one donor, while Tukey box plots represent data of all four donors. *p < 0.05, **p < 0.01

Priming of T cells with DCs pulsed with individual peptides of PP2 (P11–P20) elicited similar or greater IFN-γ T cell responses, albeit not all significant, as priming with DCs that were pulsed with the peptide pool itself (Fig. 3a). Stimulation with P11-pulsed DCs significantly increased IFN-γ production (tenfold, *p* = 0.02) to levels greater than what was observed for PP2 (sixfold, *p* = 0.04). Similarly, P12-pulsed DCs significantly increased IFN-γ production by sixfold (*p* = 0.04) compared to the control treated DCs. The strong T cell activation against P11 is also evident from the representative ELISpot image. As summarized in Fig. 3b, individual peptides P11 to P20 elicited T cell responses across multiple donors with P11 inducing the strongest response across all four donors. In comparison, although P12 induced significantly increased IFN-γ levels as depicted in Fig. 3a, only one donor D09 exhibited its strongest response against P12 and this is in conjunction with P11 and P16 (Fig. 3b). Therefore, we focused on P11 (LDHC^41−55^) in our co-culture experiments. Incubation with A2 + /high breast cancer cells specifically increased IFN-γ secretion (4.5-fold, ns) of P11-primed T cells (Fig. 3c) in comparison to T cell responses generated against A2−/high or A2 + /low breast cancer cells, confirming HLA-A*0201 restriction and suggesting the existence of a threshold for LDHC expression to elicit an immune reaction. The representative ELISpot image clearly demonstrates the increase in IFN-γ spot forming units by P11-primed T cells in co-culture with A2 + /high breast cancer cells but not with A2 + /low or A2−/high cells. In addition, we observed an increased cancer cell killing ability of A2 + /high cells by P11-primed T cells (38% versus 4% by control T cells, *p* = 0.003), whereas no significant cytolytic activity was observed against A2−/high or A2 + /low cancer cells (Fig. 3d),which is in line with the results obtained for T cell activation in Fig. 3c.

**Fig. 4:**
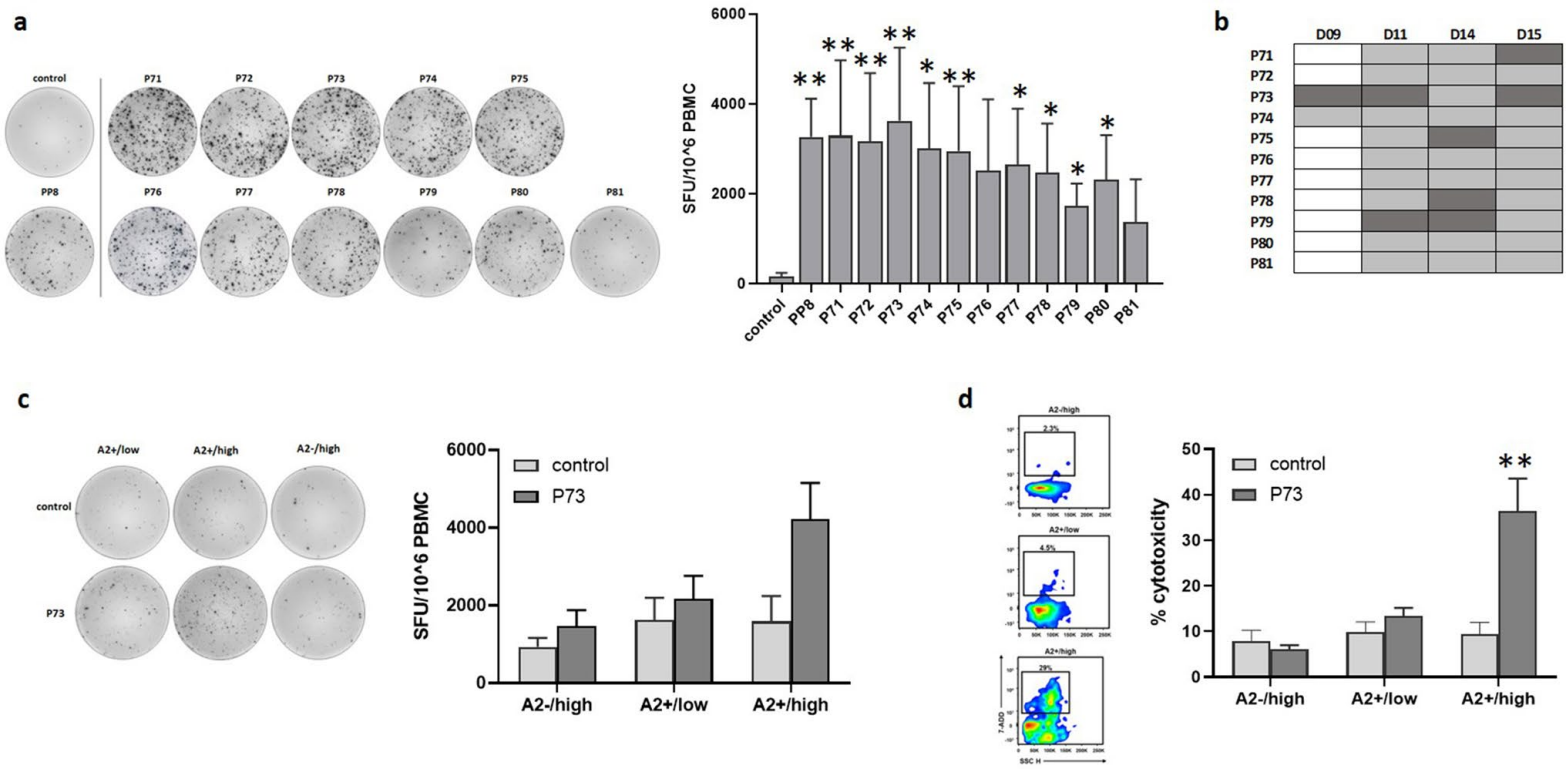
Identification of T cell responses against individual HLA-A*0201 restricted peptides within PP8. Peptide-specific T cells were generated by priming of HLA-A*0201 T cells with DCs loaded with individual peptides P71–P81 or without peptides (control). **a** T cell responses generated against individual peptides of PP8 or control (no peptides). Representative IFN-γ ELISpot images are given for one donor, while Tukey box plots represent data of all four donors. **b** Overview of T cell responses against individual PP8-peptides or control (no peptides). Positive T cell responses are depicted in grey with the strongest response for each donor highlighted in dark grey. **c** IFN-γ secretion and **d,** cytolytic activity of P73- or control (no peptides)-primed HLA-A*0201 T cells in co-culture with different breast cancer cell lines (A2 + /low, A2 + /high and A2−/high). Representative IFN-γ ELISpot images and density plots are given for one donor, while Tukey box plots represent data of all four donors. *p < 0.05, **p < 0.01

When we primed HLA-A*02 restricted T cells with DCs pulsed with individual peptides of PP8 (P71–P81), we found a wide range of significant responses (Fig. 4a). As can be seen in the representative ELISpot image, strong responses were observed against all peptides, with the weakest responses against P79 and P81. Figure 4b summarizes the results, demonstrating the variety in responses across donors with P73 (LDHC^288−303^) being the strongest inducer across three out of four donors (D09, D11 and D15). Co-culture experiments of P73-primed T cells with breast cancer cell lines showed a 2.6-fold borderline significant increase (*p* = 0.06) in IFN-γ release after incubation with A2 + /high in comparison to P73-primed cells incubated with A2 + /low (1.3-fold) or A2−/high (1.6-fold) cancer cells (Fig. 4c). The representative ELISpot image clearly shows the increase in IFN-γ spot forming units in the A2 + /high cancer cell/T cell co-culture but not in the other co-cultures. Furthermore, P73-primed T cells displayed specific cytolytic activity against A2 + /high (36% versus 9% by control T cells, *p* = 0.013) but not A2−/high or A2 + /low cancer cells (Fig. 4d). CD4 + T cell depletion following co-culture of P11- and P73-primed T cells with A2 + /high breast cancer cells revealed a significant larger IFN-γ response in the CD4 + depleted fraction in comparison to the CD4 + fraction (Supplementary Fig. 1), indicating that the observed P11- and P73-induced T cell activation and tumor cell killing (Figs. 3 and 4) are mediated by a cytotoxic CD8 + T cell response.

Together, these results suggest that LDHC peptides P11 and P73 represent endogenous peptides that are expressed by breast tumor cells and correctly presented within an HLA-A*02 context with a plausible threshold of LDHC expression for cytotoxic CD8 + T cell reactivity. Indeed, further analyses using a second breast cancer cell line model shows a strong increase in IFN-γ release and cytolytic activity of P11- and P73-primed T cells against BT549 cells with high expression of LDHC (A2 + /high) versus their counterparts after LDHC silencing (A2 + /low) (Supplementary Fig. 2).

To confirm the HLA-A*02 specificity, we predicted the HLA-peptide binding of both peptides for all HLA class I alleles using the Syfpeithi algorithm [29], which revealed a HLA-A*0201 binding score of 28 (DLADELALV nonamer) and 24 (LADELALVDV decamer) for P11 (LDHC^41−55^), and 18 (LGRNGVSDV nonamer) and 25(VLGRNGVSDV decamer) for P73 (LDHC^288−303^) (Table 2). Moreover, we assessed the HLA-A*02 specificity of P11 and P73 using T2 cells as antigen presenting cells. P11- and P73-loaded T2 cells significantly increased IFN-γ production of peptide-primed T cells, whereas no T cell activation was observed using T2 cells loaded with the non-reactive LDHC peptide P78 or no peptides control (Supplementary Fig. 3).

### Immunophenotyping of LDHC-induced T cells

**Fig. 5:**
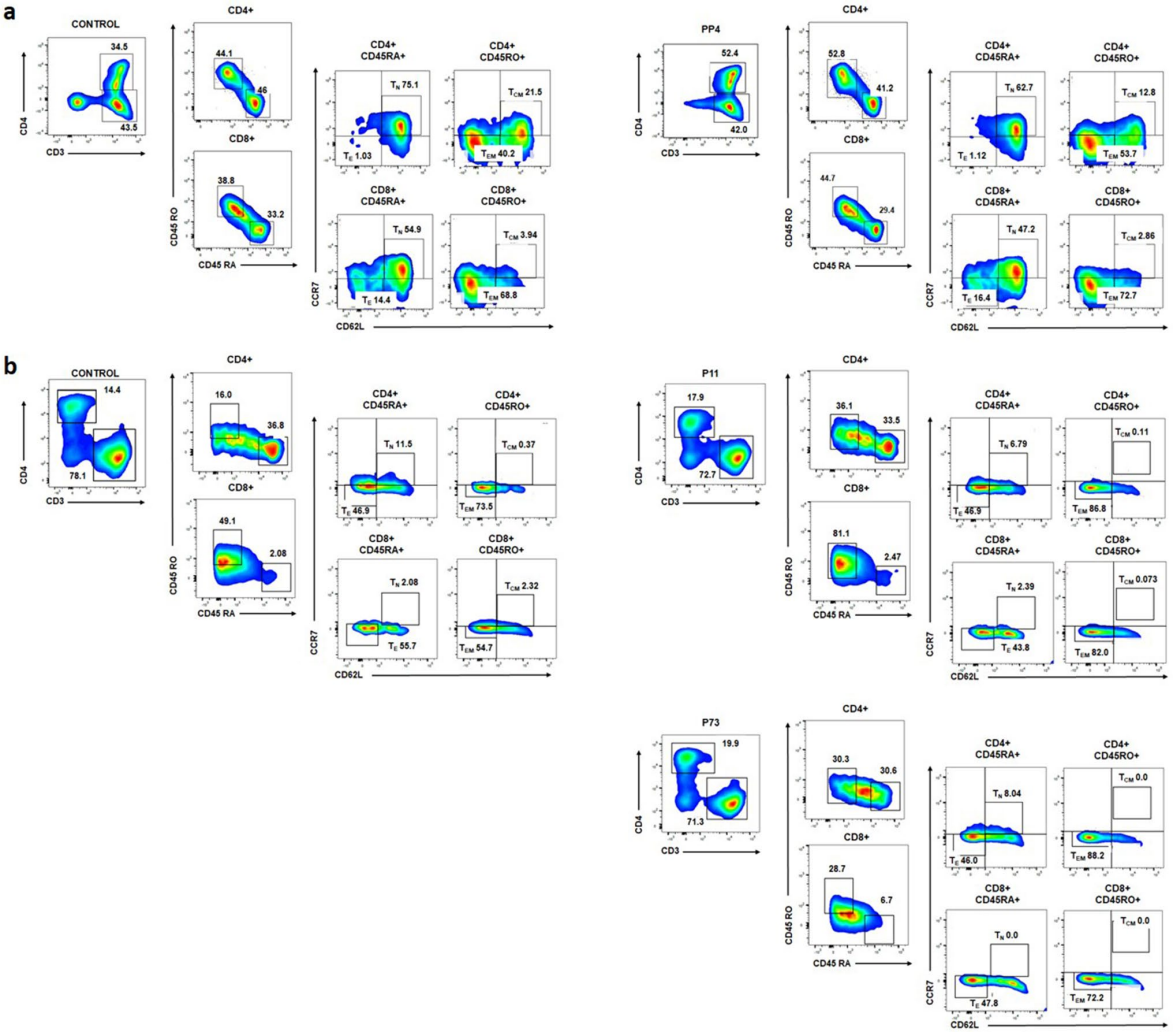
Characterization of generated T cells against LDHC-derived peptides. Multi-parameter flow cytometry was conducted to assess the frequency of CD4^+^ and CD8^+^ central memory (T_CM_), effector memory (T_EM_), naïve (T_N_), and effector (T_E_) T cells. **a** Frequency of immune cell subsets among T cell responses after in vitro stimulation with peptides or control (no peptides). Representative density plots show an increase in CD4^+^ and CD8^+^ T_EM_ cells of donor D03 after stimulation with PP4 as compared to control (no peptides)-stimulated T cells. **b** Frequency of immune cell subsets among T cell responses after priming with autologous pulsed-DCs. Representative density plots show an increase in CD4^+^ and CD8^+^ T_EM_s of donor D09 after stimulation with P11- and P73-pulsed DCs as compared to the cell subpopulations of T cells primed by control (no peptides)-pulsed DCs

Given the positive T cell responses after IVS and co-culture with A2 + /high cancer cells, we characterized the phenotype of the generated T cells. Using a multi-parameter flow cytometry analysis, we determined the frequency of CD4^+^ and CD8^+^ central memory (T_CM_), effector memory (T_EM_), naïve (T_N_) and effector (T_E_) T cells. Analysis of T cells after in vitro stimulation with peptide pools revealed an increase in mainly CD4^+^T_EM_T cells and a minor change in CD8^+^T_EM_T cells compared to the control-treated T cells. For example, when analyzing the strongest positive T cell response against PP4 in donor D03 (Fig. 1a), we found an increase in CD4^+^ T_EM_ cells (54% versus 40% in control) with a slight change in the number of CD8^+^ T_EM_ cells (73% versus 69% in control) (Fig. 5a). Likewise, priming of HLA-A*0201- restricted T cells with peptide-pulsed dendritic cells increased the CD4^+^T_EM_ and CD8^+^ T_EM_ cell population. As depicted in Fig. 5b, for instance, priming of T cells from donor D09 with P11 (LDHC^41−55^)- and P73 (LDHC^288−303^)-pulsed dendritic cells increased the number of CD4^+^ T_EM_ cells by 13–14% (74% for control, 87% for P11, and 88% for P73), and of CD8^+^T_EM_ cells by 17–26% (55% for control, 82% for P11and 72% for P73).

## Discussion

Cancer testis antigens are gaining interest as targets for adoptive T cell therapy with numerous preclinical studies and clinical trials focusing on NY-ESO-1, MAGE-A3 and PRAME [30–32]. To date, there are no published data on the immunogenicity and targetability of the cancer testis antigen LDHC for cancer immunotherapy except for a Master’s thesis that is deposited in the public domain [33]. LDHC expression has been detected in different tumor types at varying degrees with frequencies up to 100% in lung adenocarcinoma, 83% in cervical cancer, 76% in high-grade serous ovarian carcinoma (HGSC), 44% in melanoma, and 35% in breast cancer [6, 33, 34]. As aforementioned, to the best of our knowledge, the immunogenicity of LDHC has been investigated in one study only that demonstrated the presence of LDHC-peptide reactive T cells in the ascites of three of five patients with high-grade serous ovarian carcinoma [33]. Following expansion of LDHC-reactive T cells of 2 out of 3 patients; one patient displayed specific T cell responses against one peptide pool. Further analysis revealed that these responses were elicited by the 11-mer YTSWAIGLSVM peptide, corresponding to peptide p62. However, p62-specific T cells were not able to recognize autologous ascites, autologous B cells transfected with LDHC or tumor cell lines with endogenous LDHC. Of note, the 11-mer peptide identified in their study was also included in our peptide pool 7 as P62. In our study, we did not observe any strong T cell responses against P62, as determined by IFN-γ release, using in vitro peptide stimulation of T cells isolated from 14 healthy donors, rather than patients. HLA prediction analysis of the P62 peptide sequence (KGYTSWAIGLSVMDL) did not reveal strong nonamer or decamer binders for the reported HLA haplotype of the HGSC patient (HLA-A*02, HLA-B*08, HLA-B*057, HLA-C*06, and HLA-C*07) which may in part explain the lack of endogenous LDHC-reactive T cell responses in the study. In addition, it is unclear whether the LDHC-derived peptide is processed and presented as a cognate peptide and to what extent LDHC is expressed in the patient’s tumor cells and the tumor cell lines. Furthermore, it is likely that pre-existing high-affinity LDHC-specific T cells are partially exhausted or are only present in the tumor microenvironment and not in the ascites of patients.

In our study, we found a wide range of T cell responses against synthetic 15-mer LDHC-derived peptides using T cells from 14 healthy donors. Further analysis of four HLA-A*02 healthy individuals demonstrated the immunogenic potential of two individual peptides, P11 (LDHC^41−55^) and P73 (LDHC^288−303^), and the functional activity of the respective-primed T cells against breast cancer cell lines. HLA binding prediction and peptide-T2 cell experiments support the HLA-A*0201 specificity of both peptides. LDHC^41−55^ and LDHC^288−303^-primed T cells exhibited increased IFN-γ secretion and cytolytic activity against HLA-A*0201 breast cancer cell lines with endogenous LDHC expression. In contrast, no specific T cell responses were observed against a HLA-A*0201 negative breast cancer cell line. Depletion assays demonstrated a predominant CD8 + T cell response against both peptides. Moreover, our results suggest that there is a plausible threshold of LDHC expression to elicit an anti-tumor immunity since we observed attenuated T cell responses against the HLA-A*0201 breast cancer cell line following LDHC silencing. More specifically, we observed reduced IFN-γ secretion and a complete lack of cytolytic activity of T cells in co-culture with low LDHC expressing cancer cells, which was confirmed, in a second breast cancer cell line model. Furthermore, LDHA expression is not altered in LDHC high versus LDHC low expressing cancer cells (unpublished data), suggesting that the difference in peptide-specific T cell responses in LDHC high versus LDHC low expressing cells might not be affected by cross-reactivity with LDHA cognate peptides. However, future studies are required to study cross-reactivity with LDHA and LDHB in more detail and to ensure LDHC specificity of the peptide-induced cytotoxic T cell responses. Our findings are in line with previous observations of a recognition threshold for expression of the cancer testis antigen PRAME and of different signaling thresholds for CD8^+^ T cell IFN-γ secretion and acquisition of cytolytic activity [35, 36]. The potential existence of an expression threshold for an effective anti-tumor response has important implications for LDHC-specific immunotherapy. In accordance, current efforts are directed towards increasing the expression of CTAs, including NY-ESO-1 and PRAME, through combination treatment of demethylating agents and histone deacetylase inhibitors [30, 32]. Using this combination treatment, both the intra-tumor heterogeneous expression of CTAs and the expression threshold could be addressed. Interestingly, we found that the majority of P11 (LDHC^41−55^) and P73 (LDHC^288−303^) specific T cells displayed an effector memory phenotype. This is of importance since an effective long-term anti-tumor response requires multiple T cell subpopulations, including memory and effector cells. We found an increase in the number of CD4^+^ and CD8^+^T effector memory cells after priming with the immunogenic LDHC-derived peptides P11 (LDHC^41−55^) and P73 (LDHC^288−303^), and after co-culture with HLA-A*0201/LDHC positive breast cancer cells, suggesting that the CD8^+^ T effector memory cell population was responsible for the cancer cell killing in our in vitro model. Likewise, it has been reported that the majority of redirected T cells against NY-ESO-1 is comprised of CD8^+^ effector memory cells [37]. However, it might be beneficial to also expand the smaller population of LDHC-specific T central memory cells to more readily sustain in vitro proliferation and in vivo persistence after antigen re-encounter [38, 39]. Indeed, secondary activation or re-stimulation of NY-ESO-1-specific T central memory cells in vitro induced differentiation into functional effector T cells which may be able to generate an anti-tumor immune response against minimal residual disease [37].

To conclude, this is the first study to induce T cell responses against numerous LDHC-derived peptides and identified two HLA-A*0201 restricted LDHC-specific peptides, P11 (LDHC^41−55^) and P73 (LDHC^288−303^), with immunogenic potential. Moreover, we were able to demonstrate the functional activity of the peptide-specific CD8 + T cells against breast cancer cell lines with endogenous LDHC expression, albeit with a constraint of an antigen threshold. Given the expression of LDHC in breast tumors, future studies are needed to evaluate the presence of pre-existing LDHC^41−55^ and LDHC^288−303^-reactive T cells in the peripheral blood of breast cancer patients.

### Electronic supplementary material

Below is the link to the electronic supplementary material.

Supplementary file1 (PDF 565 kb)

## Abbreviations

7-AAD: 7-Aminoactinomycin D
ATCC: American Tissue Culture Collection
BCA: Bicinchoninic acid assay
CTA: Cancer testis antigen
T_CM_: Central memory T cell
D: Donor
ECL: Enhanced chemiluminescence
T_E_: Effector T cell
T_EM_: Effector memory T cell
A2−/high: HLA-A*02 negative/LDHC high expression
A2 + /low or A2 + /high: HLA-A*02 positive/LDHC low or high expression
HGSC: High-grade serous ovarian carcinoma
IVS: In vitro stimulation
LDHA/LDHB/LDHC: Lactate dehydrogenase A/B/C
MAGE-A3: Melanoma-associated antigen 3
MMLV: Moloney murine leukemia virus
MMP9: Matrix metalloproteinase 9
NY-ESO-1: New York esophageal squamous-cell carcinoma 1
T_N_: Naïve T cell
PP: Peptide pool
PRAME: Preferentially expressed antigen in melanoma
RIPA: Radioimmunoprecipitation assay
RPLPO: 60S acidic ribosomal protein P0
SFU: Spot forming units
shRNA: Short hairpin RNA
TBST: Tris-buffered saline and 0.1% tween-20
TGX: Tris-glycine extended
