# Supplementary material for: Identification of two HLA-A*0201 immunogenic epitopes of lactate dehydrogenase C (LDHC): potential novel targets for cancer immunotherapy
Source: Cancer Immunol Immunother. 2020 Jan 13;69(3):449–63. doi: 10.1007/s00262-020-02480-4 (PMC7044258; doi:10.1007/s00262-020-02480-4)
Supplement: Supplementary file 1 — Supplementary file1 (PDF 565 kb) [file 262_2020_2480_MOESM1_ESM.pdf]

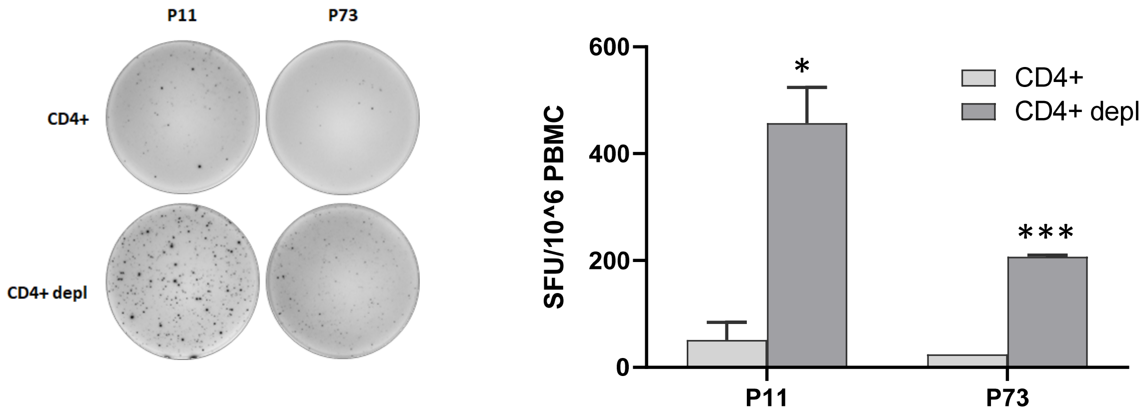

Supplementary Figure 1. CD4+ and CD4+ depleted peptide-specific T cell immune responses. Following co-culture with A2+/high HCC1500 breast cancer cells, P11- and P73-primed T cells were fractionated into 2 subpopulations, a CD4+ and CD4+depleted T cell subset, for IFN-γ ELISpot analysis. Representative IFN-γ ELISpot images are given for one donor, while Tukey box plots represent data of two donors. * $p<0.05$, *** $p<0.001$.

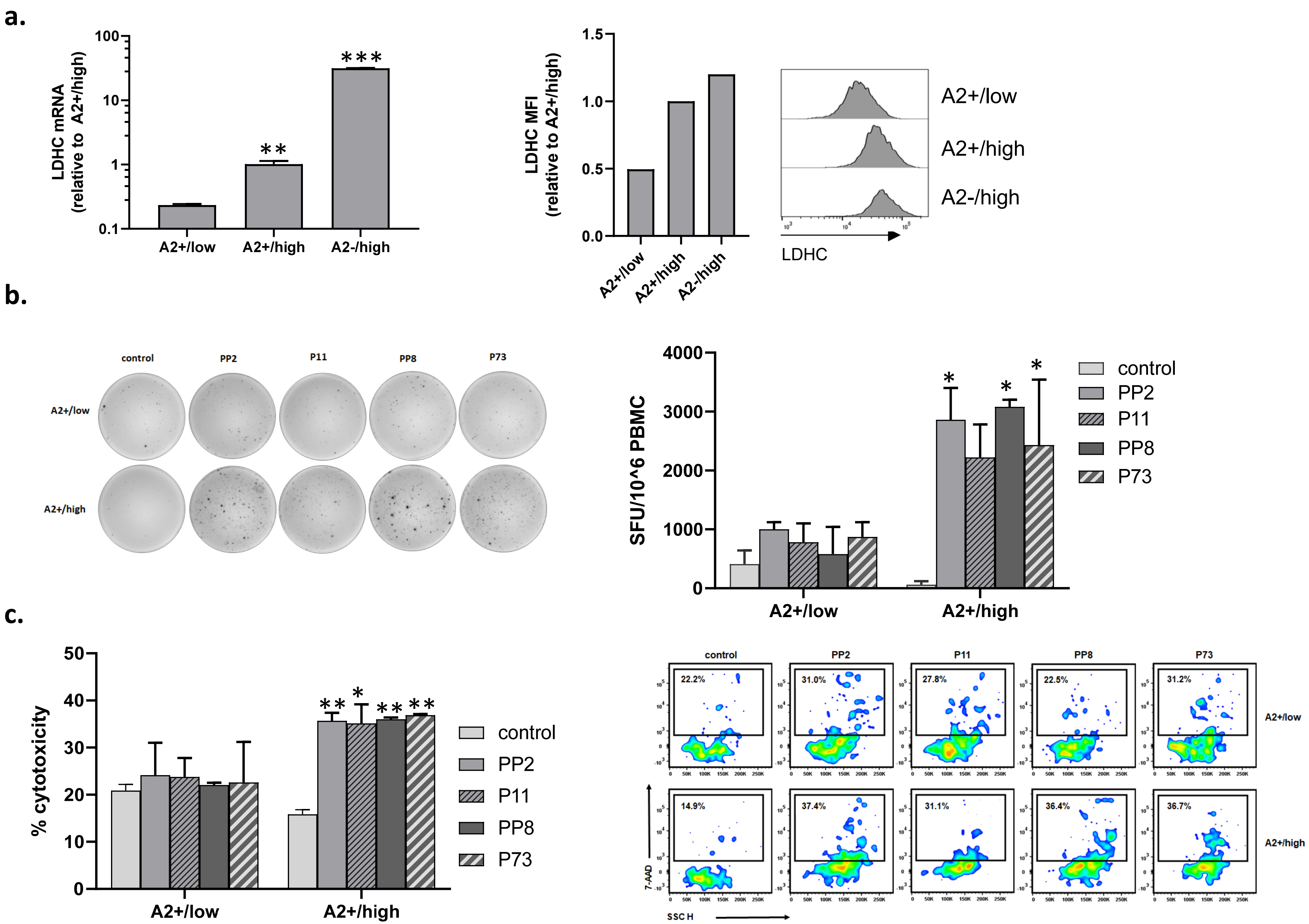

Supplementary Figure 2. Immunogenicity of P11- and P73- specific T cells against loss-of-function LDHC BT549 breast cell line model. a, LDHC expression of shCTR (A2+/high) and shLDHC (A2+/low) BT549 cells, determined by qRT-PCR, western blot and flow cytometry. **b,** IFN-γ release and **c,** cytolytic activity of PP2-, PP8- and P11- and P73-specific T cells in co-culture with A2+/high and A2+/low BT549 cells. Representative IFN-γ ELISpot images are given for one donor, while Tukey box plots represent data of two donors. MFI, mean fluorescence intensity. * p<0.05, ** p<0.01, *** p<0.001.

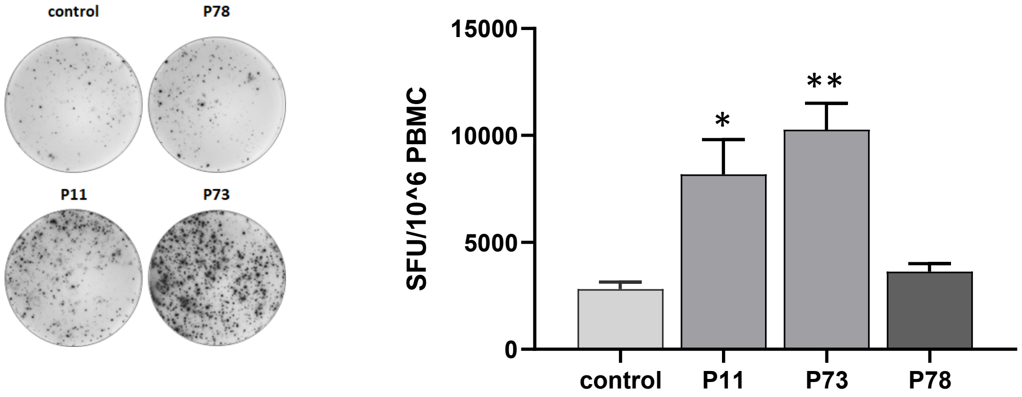

Supplementary Figure 3. HLA-A*02 specificity of P11- and P73-induced T cell responses. LDHC peptide loaded-T2 cells were incubated with their respective primed T cells followed by measurement of IFN-γ release. T2 cells were loaded with either P11, P73 or no peptide (control) in addition to a non-reactive peptide (P78). Representative IFN-γ ELISpot images are given for one donor, while Tukey box plots represent data of two donors. . * $p<0.05$, ** $p<0.01$.
